# Immune checkpoint inhibitors in *MITF* family translocation renal cell carcinomas and genetic correlates of exceptional responders

**DOI:** 10.1186/s40425-018-0482-z

**Published:** 2018-12-27

**Authors:** A. Boilève, M. I. Carlo, P. Barthélémy, S. Oudard, D. Borchiellini, M. H. Voss, S. George, C. Chevreau, J. Landman-Parker, M-D Tabone, D. D. Chism, A. Amin, M. A. Bilen, D. Bosse, A. Coulomb-L’hermine, Xiaoping Su, T. K. Choueiri, Nizar M. Tannir, Gabriel G. Malouf

**Affiliations:** 1 Department of Medical Oncology, Hôpital Universitaire Pitié-Salpétrière, Paris, France; 2 Department of Medicine, Memorial Sloan-Kettering Cancer Center, New York, NY USA; 3 Service d’Hématologie et d’Oncologie, Centre Hospitalier Universitaire de Strasbourg, Strasbourg, France; 4 Oncology Department, European Georges Pompidou Hospital, René Descartes University, Paris, France; 5 Association pour la Recherche sur les Thérapeutiques Innovantes en Cancérologie, Paris, France; 6 U790 PARCC, European Georges Pompidou Hospital, René Descartes University, Paris, France; 7 Centre Antoine Lacassagne, Nice, France; 8 Department of Medicine, Roswell Park Cancer Institute, Buffalo, NY USA; 9 IUCT-Oncopole, Institut Claudius-Regaud, Toulouse, France; 10 Service d’Hématologie et d’Oncologie Pédiatrique, Hopital Armand-Trousseau, Paris, France; 11 Division of Hematology and Oncology, Department of Medicine, Vanderbilt Ingram Cancer Center, Vanderbilt University Medical Center, Nashville, TN USA; 12 Carolinas Healthcare System, Levine Cancer Institute, Charlotte, NC USA; 13 Department of Hematology and Medical Oncology, Winship Cancer Institute of Emory University, Atlanta, GA USA; 14 Department of Medical Oncology, Dana-Farber Cancer Institute and Brigham and Women’s Hospital, Harvard Medical School, Boston, MA USA; 15 Service d’Anatomopathologie, Hôpital Armand-Trousseau, Paris, France; 16 Department of Bioinformatics and Computational Biology, The University of Texas MD Anderson Cancer Center, Houston, TX USA; 17 Department of Genitourinary Medical Oncology, The University of Texas MD Anderson Cancer Center, Houston, TX USA; 18 Department of Functional Genomics and Cancer, Institut de Génétique et de Biologie Moléculaire et Cellulaire, Illkirch, France; 19 Department of Hematology and Oncology, Centre Hospitalier Universitaire de Strasbourg, 1, Place de l’Hôpital, 67000 Strasbourg, France

**Keywords:** TFE3, TFEB, Antiangiogenic agents, O-glycosylation, Bromodomain-containing genes, Parallel evolution

## Abstract

**Background:**

*Microphthalmia Transcription Factor* (*MITF*)family translocation renal cell carcinoma (tRCC) is a rare RCC subtype harboring *TFE3*/*TFEB* translocations. The prognosis in the metastatic (m) setting is poor. Programmed death ligand-1 expression was reported in 90% of cases, prompting us to analyze the benefit of immune checkpoint inhibitors (ICI) in this population.

**Patients and methods:**

This multicenter retrospective study identified patients with *MITF* family mtRCC who had received an ICI in any of 12 referral centers in France or the USA. Response rate according to RECIST criteria, progression-free survival (PFS), and overall survival (OS) were analyzed. Genomic alterations associated with response were determined for 8 patients.

**Results:**

Overall, 24 patients with metastatic disease who received an ICI as second or later line of treatment were identified. Nineteen (82.6%) of these patients had received a VEGFR inhibitor as first-line treatment, with a median PFS of 3 months (range, 1–22 months). The median PFS for patients during first ICI treatment was 2.5 months (range, 1–40 months); 4 patients experienced partial response (16,7%) and 3 (12,5%) had stable disease. Of the patients whose genomic alterations were analyzed, two patients with mutations in bromodomain-containing genes (*PBRM1* and *BRD8*) had a clinical benefit. Resistant clones in a patient with exceptional response to ipilimumab showed loss of *BRD8* mutations and increased mutational load driven by parallel evolution affecting 17 genes (median mutations per gene, 3), which were enriched mainly for O-glycan processing (29.4%, FDR = 9.7 × 10^− 6^).

**Conclusions:**

*MITF* family tRCC is an aggressive disease with similar responses to ICIs as clear-cell RCC. Mutations in bromodomain-containing genes might be associated with clinical benefit. The unexpected observation about parallel evolution of genes involved in O-glycosylation as a mechanism of resistance to ICI warrants exploration.

## Introduction

*Microphthalmia Transcription Factor* (*MiTF*) family translocation renal cell carcinoma (tRCC) is a subtype of RCC characterized by chromosomal translocations involving *TFE3* and *TFEB* transcription factor genes [1]. As tRCCs with *TFE3* or *TFEB* mutations share clinical, histopathological and molecular features, the 2013 ISUP Vancouver classification grouped these entities as the “*MiTF/TFE* translocation carcinomas family” [2]. The frequency of adult *TFE3* tRCC has been reported to range between 1 and 5% of all RCCs [3–5]. tRCC usually occurs in children, adolescents and young adults, with a high female predominance [3–5]. There are no approved therapies for metastatic tRCC, and effective therapy for this cancer remains an unmet medical need.

The current first-line standard of care for good risk metastatic clear-cell RCC (ccRCC) is the tyrosine kinase inhibitors (TKIs) targeting vascular endothelial growth factor receptor (VEGFR) [6]. Conversely, the combination of ipilimumab and nivolumab is the standard of care for intermediate and poor risk disease [7]. While there is no standard of care for non-clear cell metastatic RCCs (referred to here as non–ccRCC), retrospective analyses indicate that VEGFR-targeted agents provide some efficacy in metastatic tRCC, with an objective response rate of 30% and a median progression-free survival (PFS) duration of 7.1–8.2 months [8, 9].

Recently, virtual karyotyping of tRCC identified a subgroup with 17q gain characterized by activation of the cytotoxic T lymphocyte–associated protein 4 (CTLA4) pathway [10]. Another study exploring programmed death ligand 1 (PD-L1) expression in a wide range of non–ccRCC identified PD-L1 overexpression in tumor-infiltrating immune cells in 90% of tRCC cases [11]. Those studies prompted us to explore the efficacy of immune checkpoint inhibitors (ICIs) in this setting. Nivolumab, a programmed death 1 (PD-1) checkpoint inhibitor, was associated with longer overall survival (OS) than mTOR inhibitors in a phase III study involving previously treated patients with metastatic ccRCC and is now often used as second-line therapy [12]. Currently, data regarding the efficacy of ICIs in non–ccRCC are limited, and results of clinical trials are pending.

The purpose of this study is to determine the efficacy of ICIs in the treatment of tRCC and to correlate tumor genomic alterations with objective response. We performed a retrospective multicenter analysis of the outcomes of patients with tRCC treated with an ICI in 12 institutions in France and the USA. The efficacy of first-line TKI treatment was also analyzed.

## Patients and methods

### Patients

Patients with tRCC were identified through searches of the patient databases of 12 institutions in France and the USA for the period from July 2011 to May 2017. Inclusion criteria included tRCC diagnosed by immunohistochemical analysis (IHC) and treatment with at least one ICI. A dedicated genitourinary pathologist at each of the participating institutions verified tRCC diagnoses. *TFE3* expression was confirmed by IHC analysis in all cases. FISH confirmation was not a requirement in this study, but was available in the majority of cases. Cases that were tested but not confirmed by FISH were excluded. Clinical characteristics and treatment-related outcome data for ICIs (targeting PD-1, PD-L1 or CTLA4), administered alone or in combination with other agents, were retrospectively determined by individual chart review. We collected data concerning prior treatments, first metastasis, date of first treatment, toxic effects, date of progression and date of death or last follow-up contact. All patients’ data were anonymized and de-identified prior to analysis. Patient data were collected in compliance with the IRB guidelines of each participating institution. Written informed consent was obtained from all patients for whom genomic testing was performed. All study protocols were performed in accordance with the ethical tenets of the Declaration of Helsinki.

### Assessment of tumor response

Patients were monitored by their physician until the end of treatment. All treatments and responses, from diagnosis to death or loss to follow-up, were recorded. Tumor response and disease progression by RECIST 1.1 criteria were documented. Stable disease was defined as a stable RECIST response for more than 3 months. Clinical benefit was defined as Miao et al. and included patients with partial response or stable disease lasting more than 6 months [13].

### Genomic analysis

Targeted sequencing data on 410 cancer genes using MSK-IMPACT were collected on tumors from 4 cases, with a median coverage of 580x per case (range, 230–1141) [14]. Whole-exome sequencing was performed on another 4 tumors and matched normal adjacent tissues. Briefly, exomes were captured using Agilent SureSelect Human All Exon 50 Mb (Agilent Technologies, Santa Clara, CA, USA) according to the manufacturer’s instructions. The technical details and mutation detection method were as previously described [15]. Median coverage obtained for tumor samples was ~100x. Mutational load was defined as the total number of somatic mutations obtained per whole-exome sequencing. To compare the mutational load of these tRCCs with mutational load in ccRCC, somatic mutations of ccRCC cases from The Cancer Genome Atlas (TCGA) were retrieved from a report on ccRCC published by TCGA [16].

### Statistical analysis

Study endpoints were response rates according to RECIST criteria PFS, and OS. The Kaplan-Meier method was used for survival analyses. PFS was measured from the date of initiation of ICI treatment to the time of progression at any site or death from any cause. All statistical analyses were done by using GraphPad Prism (GraphPad Software, La Jolla, CA, USA).

## Results

### Patient characteristics

**Fig. 1:**
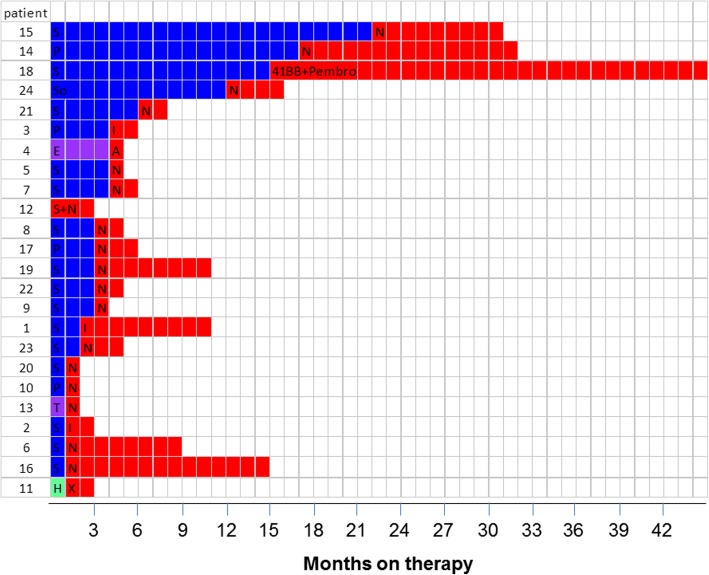
Responses to tyrosine kinase inhibitors and immune checkpoint inhibitors in 24 patients with metastatic *MITF* family translocation renal cell carcinoma. Blue/purple/cyan: first-line therapy; red: second-line therapy. S, sunitinib; P, pazopanib; So, sorafenib; N, nivolumab; I, ipilimumab; A, atezolizumab; X, combination MEDI4736 + tremelimumab; 41BB + Pembro, 41BB agonist and pembrolizumab; E, everolimus; T, temsirolimus; H, high-dose IL2; NA, Not available

Overall, we identified 24 patients who met the inclusion criteria. Selected demographic and clinical characteristics of these patients are summarized in Tables 1 and 2. Before receiving an ICI, the majority of patients had received a VEGFR-targeted agent as first-line therapy (Fig. 1).

**Table 1:** Selected baseline demographic and clinical characteristics of 24 patients with metastatic *MITF* family translocation renal cell carcinoma treated with an immune checkpoint inhibitor

Characteristics	Number of patients	Percentage of patients
Sex		
Male	4	20
Female	20	80
Age, years		
Median	34	
Range	3–79	
≤ 34	11	46
> 34	13	54
Karnofsky score		
≤ 80	9	40
> 80	13	60
Translocation type		
TFE3	21	88
TFEB	3	12
Common site of metastasis		
Lymph nodes	15	63
Lung	8	33
Liver	8	33
Bone	8	33
Heng score		
0 (favorable)	2	8
1–2 (intermediate)	18	75
3–4- 5 (poor)	4	17
First-line therapy		
Sunitinib	15	63
Pazopanib	4	17
Sorafenib	1	4
m-TOR inhibitor	2	8
High dose IL2	1	4

**Table 2:** Outcomes for 24 patients with metastatic *MITF* family translocation renal cell carcinoma treated with an immune checkpoint inhibitor (ICI)

Patient number	Sex	Age at diagnosis, years	Translocation type	TNM at diagnosis	FISH analysis	Karnofsky score	Heng score (IDMC criteria)	First treatment line	Response to 1st line	PFS (mo)	Immunotherapy treatment	ICI line	Response to ICI	Duration of response (mo)	Deseased	Survival (mo)
1	F	25	TFE B	T3N2M1	available	100	2	Sunitinib	PD	2	Ipilimumab	4	PR	9.0	no	40
2	F	3	TFE 3+	TxN0M0	available	100	1	Sunitinib	PD	1	Ipilimumab	5	PD	2.5	yes	23.5
3	F	13.7	TFE 3+	T2N + M+	available	60	3	Pazopanib	PR	4	Ipilimumab	3	PD	2,0	yes	20.7
4	F	10	TFE 3+	TxNxM0	available	< 80	1	Everolimus	PD	4.4	Atezolizumab	2	PD	0.8	no	19
5	F	35.5	TFE 3+	T2bN1Mx	available	100	4	Sunitinib	PD	4.4	Nivolumab	5	PD	1.0	yes	24.5
6	M	22	TFE 3+	pT1bNxM0	available	100	1	Sunitinib	PD	1	Nivolumab	2	PR	8.3	no	17
7	F	26.7	TFE 3+	pT1bNxMxR1	available	100	0	Sunitinib	PD	4	Nivolumab	3	PD	2.4	no	25
8	M	60.7	TFE B	pT3cN0M1R1	available	100	1	Sunitinib	PD	3.1	Nivolumab	3	PD	2.5	yes	19.5
9	F	35	TFE 3+	pT3aN1Mx	available	100	1	Sunitinib	PD	2.7	Nivolumab	2	PD	1.0	yes	9.6
10	M	41.9	TFE 3+	pT3bN1Mx	no	90	1	Pazopanib	PD	1.2	Nivolumab	2	PD	1.4	yes	8.5
11	F	35.1	TFE 3+	T3cN0M0	available	90	1	High dose IL-2	PD	0.1	MEDI4736 + Tremelimumab	4	PD	2.4	yes	16.9
12	F	16.1	TFE 3+	T1aN1M1	available	70	2	Sunitinib +nivolumab	SD	3.2	Sunitinib +nivolumab	1	PR	3.2	yes	7.6
13	F	79.5	TFE 3+	T3aN0M0	available	60	1	Temsirolimus	SD	1.2	Nivolumab	5	PD	1.0	yes	17.3
14	F	54	TFE 3+	T2aN0M1	available	80	2	Pazopanib	PR	17.2	Nivolumab	2	SD	15.4	no	84.6
15	F	42.8	TFE 3+	T3N + M+	available	100	1	Sunitinib	SD	22	Nivolumab	4	PD	9.0	no	59
16	M	32	TFE B	T3bN0M1	available	80	0	Sunitinib	PD	1	Nivolumab	2	SD	14	no	25
17	F	29.4	TFE 3+	T4N1M1	available	80	5	Pazopanib	SD	3	Nivolumab	2	PD	3	no	4
18	F	26.7	TFE 3+	NA	no	90	2	Sunitinib	SD	15	pembro +41BB agonist	3	PR	30	yes	60
19	F	49	TFE 3+	pT4pN1pM1	no	NA	1	Sunitinib	PD	3	Nivolumab	2	SD	8.5	yes	19
20	F	43	TFE 3+	T3bN0M0	available	90	1	Sunitinib	PD	1.5	Nivolumab	3	PD	1	yes	8
21	F	63	TFE 3+	pT3aNxMx	available	90	1	Sunitinib	PD	6	Nivolumab	5	PD	2	yes	24
22	F	48.2	TFE 3+	T3NxM1	available	70	3	Sunitinib	PD	3	Nivolumab	2	PD	2	no	5
23	F	45	TFE 3+	pT3aNxM1R1	available	80	1	Sunitinib	PD	2	Nivolumab	2	PD	3	yes	9
24	F	24	TFE 3+	pT2bN1	available	NA	1	Sorafenib	SD	12	Nivolumab	3	PD	4	no	70

PR: partial response; SD: stable disease; PD: progressive disease

### Clinical outcomes: First-line VEGFR-targeted agents

**Fig. 2:**
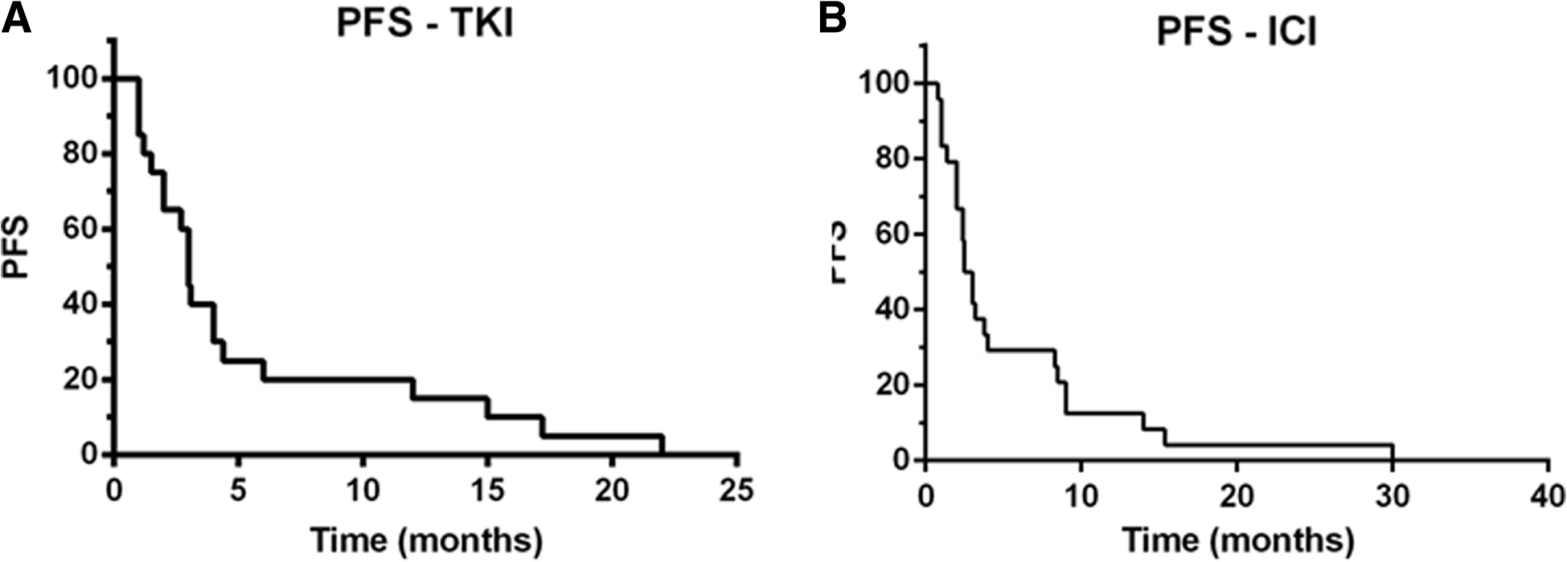
Kaplan-Meier curves for progression-free survival (PFS) of patients with metastatic *MITF* family translocation renal cell carcinoma treated with (**a**) a tyrosine kinase inhibitor (TKI) or (**b**) an immune checkpoint inhibitor (ICI)

Median PFS for first-line TKI therapy was 3 months (range, 1–22 months) (Fig. 2a). Partial responses were observed in 2 patients (10.5%), and 15 patients exhibited disease progression at the time of the first interim assessment. Six patients received an mTOR inhibitor (2, first line; 4, second line or later) and none achieved objective response. The toxic effects of sunitinib, the most frequently received first-line agent (*n* = 15), were comparable overall to those reported in studies in RCC and included mainly asthenia and rash.

### Clinical outcomes: First immune checkpoint inhibitor

Of the 24 patients, 17 received nivolumab, 3 received ipilimumab and 4 received ICI-based combination therapy (Table 2). All patients received at least one dose of an ICI; 22 (91.6%) received 4 doses or more. The median PFS was 2.5 months (range, 1–40 months) (Fig. 2b). Four patients (16,6%) experienced a partial response and 3 (12,5%) had stable disease in response to the ICI. Among the four patients who achieved an objective response, one received pembrolizumab in combination with a 41BB agonist [17] (PFS 30 months), two received nivolumab (PFS 8 and 3 months) and one received ipilimumab (PFS 9 months). Remarkably, one of the 5 responders, patient 1, showed partial response to ipilimumab lasting for 9 months. At the time of ipilimumab administration, this patient had an ECOG performance status (PS) of 3, with peritoneal, liver and lung metastases. His ECOG PS improved quickly on ipilimumab therapy, leading to a complete response of his abdomen and lung metastases; a residual 6 cm mediastinal mass was resected. The patient achieved partial response 4 months after starting ipilimumab, but developed bilateral grade 4 optic neuropathy, as previously described [14]. Upon progression, he began treatment with nivolumab, but 6 weeks later his disease had progressed, including development of 8 metastatic lesions in the brain. Genomic evolution of the tumor of this exceptional responder is reported below. The most frequent toxic effects of the ICIs, except for patient 1, were asthenia grade 2 (*n* = 9) and dyspnea grade 2 (*n* = 3). With a median follow-up duration of 19.3 months, the median OS was 24 months. Of note, no pseudoprogression was observed among the 24 patients.

### Genomic correlates of response to ICI

Tumor genomic was available in 8 patients treated with ICIs, four had whole exome sequencing and four targeted sequencing. Four of these patients (50%) derived clinical benefit from the ICI, including 2 patients with partial response and 2 patients with stable disease. Median interval time between NGS and start of TKI was 3.8 months (range: 0.4–50 months).

**Fig. 3:**
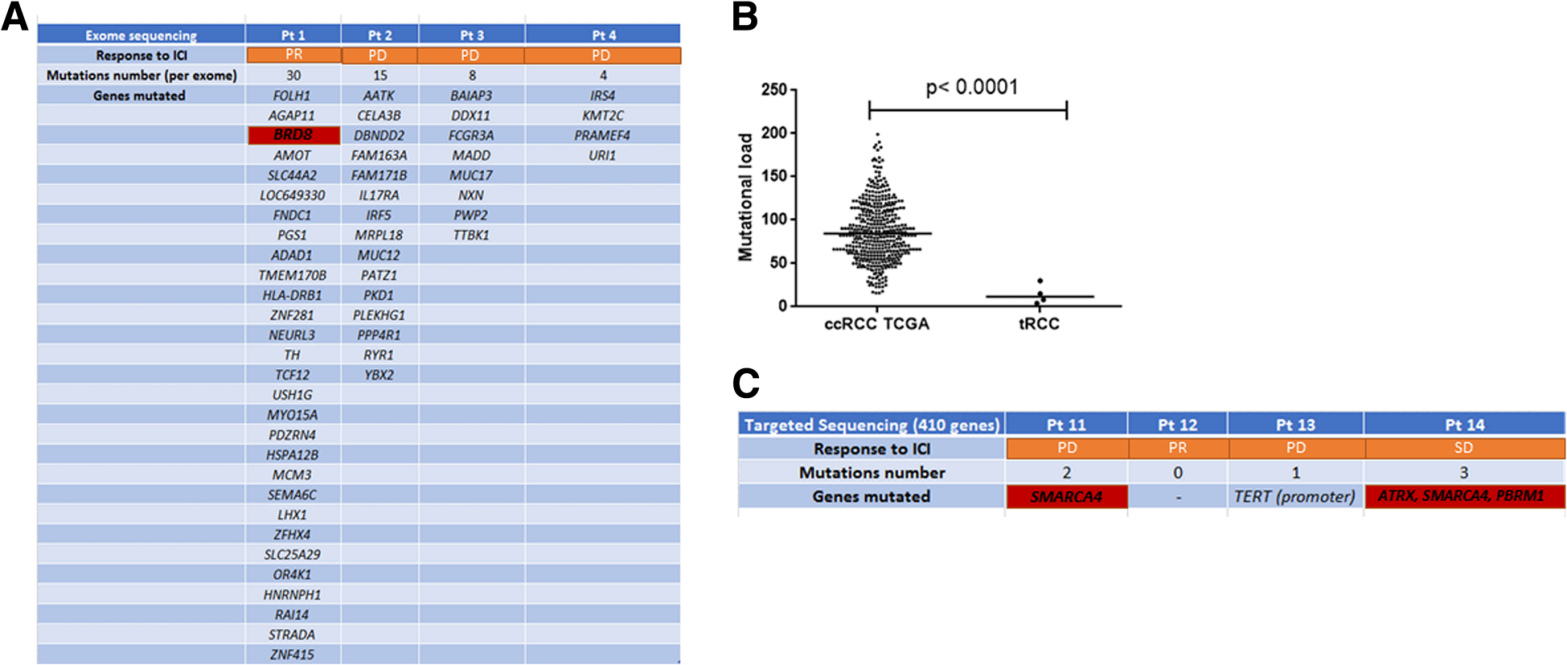
Genomic correlates of response to immune checkpoint inhibitor (ICI) therapy in a subset of 8 patients with metastatic *MITF* family translocation renal cell carcinoma (tRCC). The identified mutations and mutational load were assessed by either whole-exome sequencing or targeted sequencing. Numbers of mutations and genes mutated in each sample are given. **a** Genes mutated in the 4 samples assessed by whole-exome sequencing. **b** Box-plots depicting mutational load in tRCC patients (*n* = 4) assessed by whole-exome sequencing as compared to that in patients with clear-cell RCC (ccRCC) from the TCGA dataset (*n* = 420). **c** Genes mutated in the 4 samples assessed by targeted sequencing

The mutational load of the 4 tumors assessed by whole exome sequencing was low, ranging from 4 to 30 mutations per exome. No recurrent mutation was identified by exome sequencing (Fig. 3a). Overall, the median mutational load of these 4 tRCCs was lower than that of the ccRCC samples from the TCGA dataset (*n* = 424; *p* < 0.0001) (Fig. 3b). Focusing on the 410 cancer genes covered by both MSK-IMPACT and whole-exome sequencing in all samples, the median mutation rate in the 8 tumors was 0 (range, 0–3). Notably, *SMARCA4* mutation was the sole recurrent mutation, identified in 2 cases. The two patients which showed clinical benefit lasting for at least 6 months harbored mutations of bromodomain member genes (*PBRM1* and *BRD8*) (Fig. 3c), consistent with a recently reported association between mutations of bromodomain genes response to ICIs [18].

### Genomic landscape of resistant clones in a patient with exceptional response

**Fig. 4:**
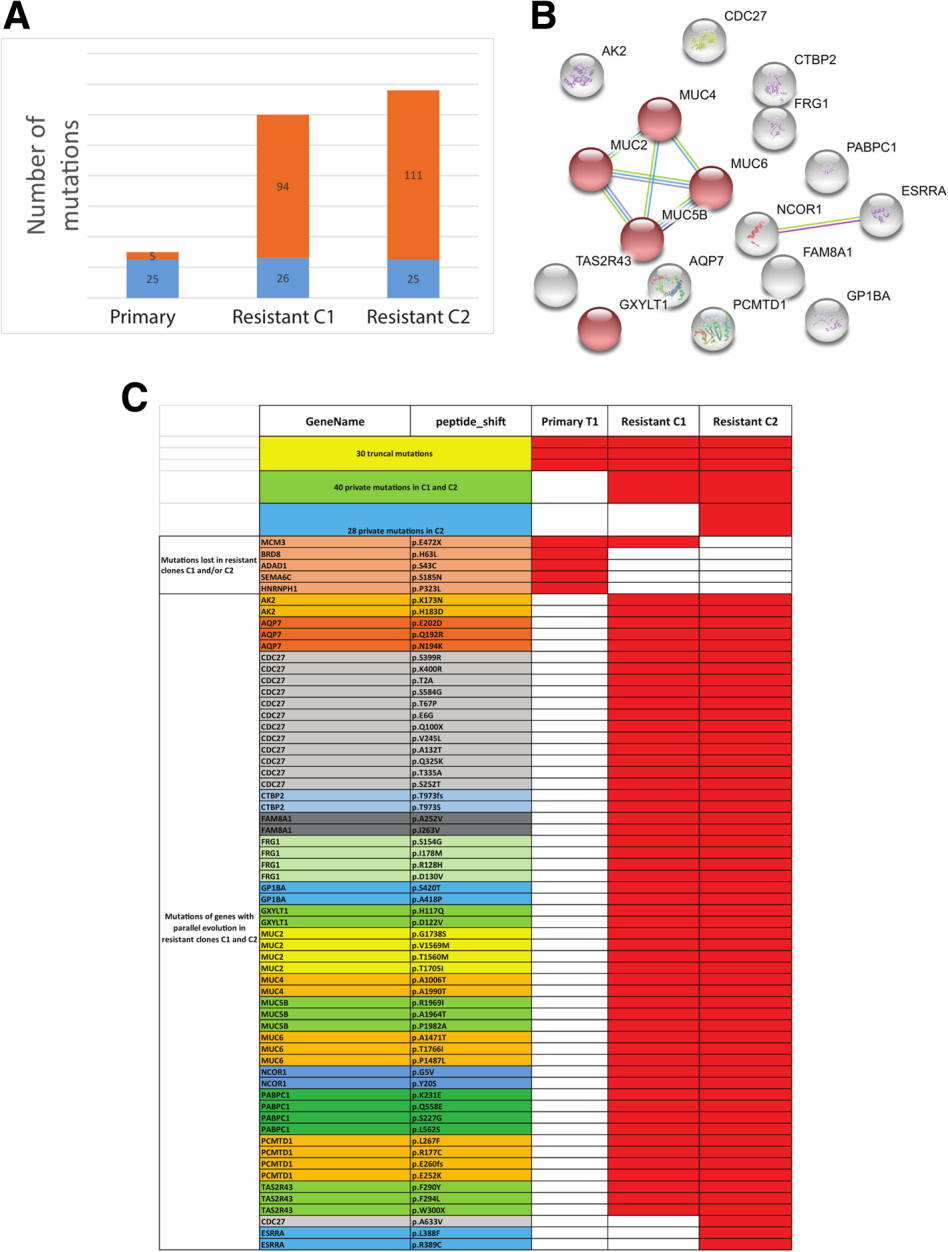
Genomic evolution of a tumor from a patient who had an exceptional response to ipilimumab. **a** Numbers of somatic mutations in the primary tumor and in the 2 resistant clones following ipilimumab treatment reveal an increase of mutational load. Blue indicates shared mutations across all 3 samples; orange indicates private mutations. **b** String network analysis of 17 genes showing parallel evolution reveals 5 genes (in red) linked to the O-glycosylation process. **c** List of somatic mutations in the primary tumor and resistant clones showing mutations lost in resistant clones as compared to primary tumor and mutations in genes with parallel evolution

As already described, patient 1 developed a dramatic response to ipilimumab lasting for 9 months; the patient had a complete response except for one resistant clone that was stable under treatment with ipilimumab, which was resected 9 months after the last ipilimumab administration and subjected to whole-exome sequencing at 2 distinct opposite regions. The number of somatic mutations in these 2 resistant clones was high, ranging from 120 to 136 mutations/50 Mb as compared to 30 mutations/50 Mb in the primary tumor (Fig. 4a). The majority of mutations present in the primary tumor (*n* = 25; 83.3%) were also present in both resistant clones, suggesting branched tumor evolution; surprisingly, the *BRD8* mutation was lost in both resistant clones. Unexpectedly, we also discovered a phenomenon of parallel evolution of somatic mutations involving 17 distinct genes, with a median of 3 somatic mutations per gene (range, 2–13) (Fig. 4b-c). Gene Ontology analysis using String identified enrichment of O-glycan processing genes (*n* = 5; false discovery rate = 9.7 × 10^− 6^) (Fig. 4b), strongly suggesting the importance of this pathway in the acquired resistance to ICI in this exceptional responder. *CDC27* was the most frequently mutated gene, involving 13 and 14 single-nucleotide polymorphisms in resistant clones 1 and 2, respectively (Fig. 4c).

## Discussion

In this international, multicenter retrospective study of 24 patients with metastatic *MITF* family tRCC who received ICI therapy, we found that 16,7% of patients had a clinical response to an ICI, with a disease control rate of 29% when stable disease was also included. Although genetic assessment was available for limited number of samples, we discovered that tumors of patients with clinical benefit harbored mutations in bromodomain-containing genes. This is, to our knowledge, the first assessment of the clinical efficacy of ICIs in patients with this type of RCC.

The lack of standard treatment for patients with metastatic tRCC is due mainly to the exclusion of patients with non–ccRCC from most large randomized trials; only a few small trials have included tRCC patients, all grouped with non–ccRCCs. Given the benefits of nivolumab in ccRCC, and the lack of other effective therapies for non–ccRCCs, this ICI is being used increasingly in non–ccRCC, although with few data to support its efficacy. Nivolumab is approved in the second-line setting for patients with RCC who have received a VEGFR-targeted agent, based on the results of Checkmate 025, a randomized phase III trial comparing nivolumab to everolimus [12]. Patients treated with nivolumab had a longer OS (25.0 vs 19.6 months) and greater response rate (25% vs 5%), although no difference in PFS was observed. However, no patients with non–ccRCC were included in that study.

Some preliminary data support the use of ICIs in non-ccRCC. Choueiri et al. reported a series of patients with non–ccRCC whose tumors and tumor-infiltrating mononuclear cells were analyzed for PD-L1 by IHC [11]. Of the 10 patients with tRCC, 3 were shown to have PD-L1+ tumor cells and 9 PD-L1+ tumor-infiltrating cells. Two small retrospective series have reported on a combined 81 patients with non–ccRCC treated with an ICI [19, 20]. Although only 4 patients with tRCC were included in those studies, one patient had a partial response, one had stable disease, and 2 had progressive disease.

Our study considerably expands what is known about the outcomes of ICI therapy for metastatic tRCC patients. As expected, most of the patients we identified (71%) were treated with nivolumab. These patients’ median PFS, 3 months, was shorter than the 4.6 months reported for CheckMate 025, although it is generally understood that PFS is not an optimal measure to gauge benefit from nivolumab therapy [12]. Similarly, overall response rate was 16,7%, compared to 25% in CheckMate 025. To date, no predictive biomarkers have been approved for selecting RCC patients who will best respond to ICIs, although several markers have been explored [21]. Higher tumor mutational load has been correlated with response to ICIs in several tumor types [22, 23]. Our data showing a low mutational load in tRCC confirmed previous reports; the limited mutational load in tRCC, even in metastatic cases, suggests low numbers of neoantigens in these tumors. The retrospective nature and small sample size of this analysis precludes any conclusions of the predictive value for any genomic event. It is, however, important to highlight here that the two patients lasting clinical benefit harbored somatic mutations of bromodomain-containing genes PBRM1 and BRD8. Recently, mutations of PBRM1 have been shown to be associated with benefit from nivolumab in patients with ccRCC [13]. Interestingly, one of the responder received pembrolizumab in combination with a 41BB agonist, a costimulatory molecule induced upon TCR activation that promotes cell survival and enhances cytotoxic T-cell responses. This combination may have enhanced the efficacy of pembrolizumab.

Notably, this is the first published report, to our knowledge, not only of a loss of *BRD8* mutation in the 2 resistant clones in response to an ICI but also of an increase in mutational load and a phenomenon of parallel evolution affecting genes involved in O-glycosylation. Parallel evolution is a mechanism that has been demonstrated in bacteria and plants and is thought to contribute to the selection of key forces that help predict and prepare for the organism’s future evolutionary course [24]. Given the major role of glycosylation in adaptive immune activation [25], further studies are needed to clarify the importance of this process in ICI response. Furthermore, unbiased genomic screens showed recently that dysfunction of *CDC27*, a member of the anaphase-promoting complex/cyclosome, limits excessive instability of cancer chromosomes, allowing tumor cells to dynamically improve their fitness during cancer evolution [26]. Notably, the high rate of somatic mutations found in the *CDC27* gene suggests that this might provide a selective advantage, improving fitness and limiting genetic instability. Reporting genomic results of exceptional responders to immunotherapy have been shown to provide much information to explore mechanisms of immunotherapy sensitivity and resistance. For example, *PTEN* mutation and reduced expression of genes encoding neoantigens was recently identified as potential mediators of resistance to immune checkpoint therapy in one patient with metastatic uterine leiomyosarcoma who had experienced complete tumor remission for > 2 years on anti-PD-1 monotherapy [27]. In addition, long term responses to anti-PD1 immunotherapy was recently described in four patients with small cell carcinoma of the ovary, a highly aggressive monogenic cancer driven by *SMARCA4* mutations [28]; this was unexpected for a low mutation burden cancer, but the majority of the tumors demonstrated PD-L1 expression with strong associated T-cell infiltration [28].

The majority of the patients in our series received a VEGFR-targeted agent as first-line therapy prior to the ICI, with disappointing results. Two small retrospective series have specifically looked at response to VEGFR-targeted agents in tRCC [8, 9]. In one series of patients with metastatic tRCC treated with a VEGFR- or mTOR-targeted agent, the median PFS of the 21 patients who received sunitinib was 8.2 months (95% confidence interval, 2.6–14.7) [9]. In another series of 15 patients treated with a variety of VEGFR-targeted agents, the median PFS was 7.1 months, with 3 achieving a partial response [8]. The median PFS durations in these studies were considerably longer than that in our cohort. Although the small numbers of patients limit comparison, the earlier studies, which used *TFE3* staining to confirm the diagnosis, may have included patients without a true translocation, whereas in this study the majority of cases (87.5%) were confirmed by FISH confirmation of translocation. Given that VEGFR-targeted therapies are still used as first-line treatment for RCC, further studies should be conducted to confirm the efficacy of these agents with molecular or FISH correlation of translocation.

Despite being one of the largest retrospective reviews, the small number of patients is the main limitation of our study. The small cohort is partly explained by the rarity of this subtype of RCC. Another limitation is that our cohort included patients with different ages at onset who received different ICIs and combinations. However, it is the first multicenter study of consecutive patients treated in several centers of expertise across Europe and the USA.

## Conclusion

In summary, ICI showed objective response in TRCC similar to those observed in clear-cell RCC. New studies are needed to explore factors associated with resistance in this setting. Mutations in bromodomain-containing genes might predict response to ICIs as reported in other cancer subtypes, and this requires prospective exploration. Importantly, responses to VEGFR-targeted agents also appear to be limited in this subtype, with a shorter PFS than previously reported, and a few durable responses were seen with ipilimumab or combination therapies [18, 20]. Given the early data showing high rates of response to combinations of an ICI and a VEGFR-targeted agent in patients with ccRCC, combinations are now being explored in clinical trials in non–ccRCC, including tRCC [NCT02724878, NCT02496208]. When available and due to rarity of this population, these trials should be considered for patients with *MITF* family tRCC. Development and studies of novel, biology-driven agents are crucially needed.

## Abbreviations

ccRCC: Clear-cell renal cell carcinoma
CTLA4: Cytotoxic T lymphocyte–associated protein 4
ICI: Immune checkpoint inhibitors
IHC: Immunohistochemical analysis
m: Metastatic
non–ccRCC: Non clear-cell renal cell carcinoma
OS: Overall survival
PD-1: Programmed death 1
PD-L1: Programmed death ligand 1
PFS: Progression-free survival
PS: Performance status
RCC: Renal cell carcinoma
TCGA: The Cancer Genome Atlas
TKI: Tyrosine kinase inhibitors
tRCC: Translocation renal cell carcinoma
VEGFR: Vascular endothelial growth factor receptor
